# Experiences of nurses responding to the COVID-19 outbreak at a long-term care hospital in Korea: A qualitative study

**DOI:** 10.1017/ash.2022.154

**Published:** 2022-05-16

**Authors:** EunJo Kim, JaHyun Kang

## Abstract

**Background:** The COVID-19 pandemic revealed the fundamental vulnerability of long-term care hospitals (LTCHs) related to infection control and prevention (ICP). We examined the experiences of nurses who worked at a hospital where a COVID-19 outbreak occurred from February 24 to March 16, 2021. **Method:** This qualitative research was performed with 9 nurses who were engaged during the COVID-19 outbreak. We prepared a semistructured questionnaire based on the main question, “How was the experience among the nurses during the outbreak, and what difficulties did they encounter while resolving the situation?” The data were collected through in-depth, individual interviews from May to August 2021 after the approval of the institutional review board, and the results were analyzed thematically. **Results:** The average age of the participants was 52.1 years, and they had an average of 15.2 years of clinical experience. We extracted 4 themes and 16 subthemes from the results. The first theme, “sudden onset of the outbreak,” included the following subthemes: (1) found myself accustomed to COVID-19 and desensitized; (2) unavoidable occurrence despite compliance with ICP guidelines; (3) LTCHs are gradually recognized as a breeding ground for COVID-19 by the public; and (4) fear of spreading the infection in the hospital and of becoming a spreader. The second theme, “heavier workload,” included (1) daily overtime and extra shifts in violation of self-quarantine recommendations due to the shortage of nurses; (2) a barrage of phone calls from family members, other departments, public health centers, and hospitals where confirmed cases were transferred; (3) nursing assistants and private caregivers who do not have ICP knowledge as well as patients who do not cooperate due to cognitive impairment; and (4) accomplishing additional tasks while wearing personal protective equipment with some suffocation. The third theme, “emotions and lessons,” included (1) unsatisfied with the initial responses; (2) awareness of the entire infectious disease; (3) increased compassion and attachment for patients; and (4) take pride in the job and the profession as a nurse. The fourth theme, “necessary support and attention,” included (1) need to install isolation rooms and replenish infection control supplies; (2) need for ICP specialists in LTCHs; (3) need for continuous national-based monitoring on ICP for LTCHs; and (4) need to improve working environment and acknowledge nurses in LTCHs. **Conclusions:** Overall, participants expressed their experiences with the insufficient infection control and response system toward COVID-19 in the LTCH. To enhance ICP in LTCHs, customized policies, regulations, and financial support for infection control activities and ICP professionals must be established.

**Funding:** None

**Disclosures:** None

